# Dimethyl 6*H*,12*H*-5,11-methano­dibenzo[*b*,*f*][1,5]diazo­cine-2,8-diacetate

**DOI:** 10.1107/S1600536808001918

**Published:** 2008-01-23

**Authors:** Masoud Faroughi, Paul Jensen, Andrew C. Try

**Affiliations:** a Department of Chemistry and Biomolecular Sciences, Building F7B, Macquarie University, NSW 2109, Australia; b Crystal Structure Analysis Facility, School of Chemistry, F11, University of Sydney, NSW 2006, Australia; c Department of Chemistry and Biomolecular Sciences, Building F7B, Macquarie University, NSW 2109, Australia

## Abstract

The asymmetric unit of the title compound, C_21_H_22_N_2_O_4_, a Tröger’s base analogue derived from methyl 4-amino­phenyl­acetate, contains two crystallographically independent mol­ecules with dihedral angles of 88.44 (5) and 88.68 (6)° between the two benzene rings.

## Related literature

For related literature, see: Faroughi *et al.* (2006 ▶, 2007 ▶, 2008*a* ▶,*b* ▶); Solano *et al.* (2005 ▶); Bag & Maitra (1995 ▶).
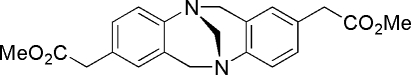


## Experimental

### 

#### Crystal data

C_21_H_22_N_2_O_4_
*M* *_r_* = 366.41
Monoclinic, 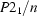

*a* = 11.559 (1) Å
*b* = 10.957 (1) Å
*c* = 28.976 (3) Å
β = 100.080 (1)°
*V* = 3613.2 (6) Å^3^
*Z* = 8
Mo *K*α radiation
μ = 0.09 mm^−1^
*T* = 150 (2) K
0.50 × 0.39 × 0.36 mm

#### Data collection

Bruker SMART 1000 CCD area-detector diffractometer
Absorption correction: multi-scan (*SADABS*; Sheldrick, 1996 ▶) *T*_min_ = 0.919, *T*_max_ = 0.967
35113 measured reflections
8689 independent reflections
5732 reflections with *I* > 2σ(*I*)
*R*_int_ = 0.036

#### Refinement

*R*[*F* ^2^ > 2σ(*F* ^2^)] = 0.047
*wR*(*F* ^2^) = 0.138
*S* = 1.01
8689 reflections
491 parameters
H-atom parameters constrained
Δρ_max_ = 0.44 e Å^−3^
Δρ_min_ = −0.22 e Å^−3^

### 

Data collection: *SMART* (Bruker, 1998 ▶); cell refinement: *SAINT* (Bruker, 2003 ▶); data reduction: *SAINT*; program(s) used to solve structure: *SHELXS97* (Sheldrick, 2008 ▶); program(s) used to refine structure: *SHELXL97* (Sheldrick, 2008 ▶); molecular graphics: *X-SEED* (Barbour, 2001 ▶) and *SHELXTL* (Sheldrick, 2008 ▶); software used to prepare material for publication: *modiCIFer* (Guzei, 2005 ▶).

## Supplementary Material

Crystal structure: contains datablocks global, I. DOI: 10.1107/S1600536808001918/hk2419sup1.cif

Structure factors: contains datablocks I. DOI: 10.1107/S1600536808001918/hk2419Isup2.hkl

Additional supplementary materials:  crystallographic information; 3D view; checkCIF report

## Figures and Tables

**Fig. 1.**
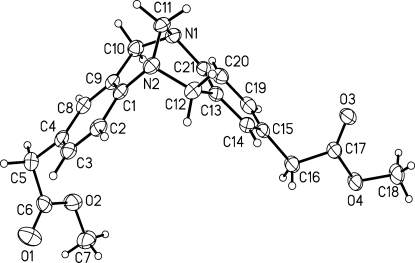
View of one of the two unique molecules present in the asymmetric unit of (I), with the atom-numbering scheme. Displacement ellipsoids are drawn at the 50% probability level.

**Fig. 2.**
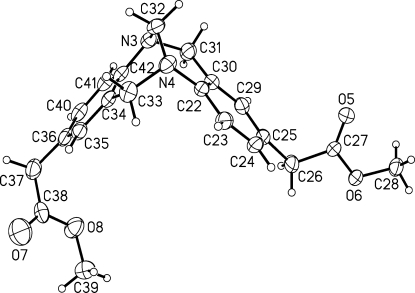
View of the second of the two unique molecules present in the asymmetric unit of (I), with the atom-numbering scheme. Displacement ellipsoids are drawn at the 50% probability level.

**Fig. 3.**
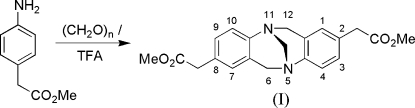
Synthetic scheme for the synthesis of (I).

## supplementary crystallographic information

### Comment

The near perpendicular arrangement of the aryl rings in Tröger's base analogues is a result of the methano-strap that is connected to the two nitrogen atoms in the diazocine bridge. Changing the length of this strap has significant effects on the geometry of the resultant compounds, with straps of three and four atoms creating a larger cavity (Faroughi *et al.*, 2007) and a strap of two atoms creating a smaller cavity (Faroughi *et al.*, 2007, 2008*a*,b). However, even within the methano-strapped family of simple dibenzo Tröger's base analogues there is significant variation of 26° in the dihedral angle that has been measured to lie between 82° (Solano *et al.*, 2005) and 108.44 (4)° (Faroughi *et al.*, 2006). Both types of molecules in the asymmetric unit of (I) shown in Fig. 1 lie toward the middle of this range, with dihedral angles of 91.56 (5)° and 91.32 (6)°.

### Experimental

The title compound was prepared according to the literature procedure (Bag & Maitra, 1995). For the preparation of the title compound, methyl 4-aminophenyl- acetate (4.14 g, 25.1 mmol) and paraformaldehyde (1.21 mg, 40.16 mmol) were dissolved in trifluoroacetic acid (50 ml) and the mixture was stirred under an argon atmosphere in the dark for 8 d. The reaction mixture was then treated with a solution of concentrated ammonia (55 ml) in water (100 ml) and further basified by the addition of a saturated sodium hydrogen carbonate solution (150 ml). The crude material was extracted into dichloromethane (3 *x* 75 ml) and the combined organic layers were washed with brine (100 ml), dried over anhydrous sodium sulfate, filtered and evaporated to dryness to yield brown solid. The crude material was chromatographed (silica gel, ethyl acetate:dichloromethane 1:3) to afford the title compound, (I), (2.82 g, 61%) as a white solid. Single crystals of (I) were produced from slow evaporation of a dichloromethane solution.

### Refinement

H atoms were positioned geometrically, with C—H = 0.95, 0.99 and 0.98 Å for aromatic, methylene, and methyl H atoms, respectively, and constrained to ride on their parent atoms, with *U*_iso_(H) = *xU*_eq_(C), where *x* = 1.5 for methyl and 1.2 for all other H atoms. The methyl groups were free to rotate about the C—O bonds.

### Crystal data

**Table:**

C_21_H_22_N_2_O_4_	*F*_000_ = 1552
*M**_r_* = 366.41	*D*_x_ = 1.347 Mg m^−^^3^
Monoclinic, *P*2_1_/*n*	Melting point: 395 K
Hall symbol: -P 2yn	Mo *K*α radiation λ = 0.71073 Å
*a* = 11.559 (1) Å	Cell parameters from 7374 reflections
*b* = 10.957 (1) Å	θ = 2.3–28.3º
*c* = 28.976 (3) Å	µ = 0.09 mm^−^^1^
β = 100.080 (1)º	*T* = 150 (2) K
*V* = 3613.2 (6) Å^3^	Prism, colorless
*Z* = 8	0.50 × 0.39 × 0.36 mm

### Data collection

**Table:**

Bruker SMART 1000 CCD area-detector diffractometer	8689 independent reflections
Radiation source: fine-focus sealed tube	5732 reflections with *I* > 2σ(*I*)
Monochromator: graphite	*R*_int_ = 0.036
*T* = 150(2) K	θ_max_ = 28.4º
ω scans	θ_min_ = 1.8º
Absorption correction: multi-scan(SADABS; Sheldrick, 1996)	*h* = −15→15
*T*_min_ = 0.919, *T*_max_ = 0.967	*k* = −14→14
35113 measured reflections	*l* = −37→37

### Refinement

**Table:**

Refinement on *F*^2^	Secondary atom site location: difference Fourier map
Least-squares matrix: full	Hydrogen site location: inferred from neighbouring sites
*R*[*F*^2^ > 2σ(*F*^2^)] = 0.047	H-atom parameters constrained
*wR*(*F*^2^) = 0.138	*w* = 1/[σ^2^(*F*_o_^2^) + (0.0601*P*)^2^ + 1.5095*P*] where *P* = (*F*_o_^2^ + 2*F*_c_^2^)/3
*S* = 1.01	(Δ/σ)_max_ < 0.001
8689 reflections	Δρ_max_ = 0.44 e Å^−^^3^
491 parameters	Δρ_min_ = −0.22 e Å^−^^3^
Primary atom site location: structure-invariant direct methods	Extinction correction: none

### Special details

**Table:**

Geometry. All e.s.d.'s (except the e.s.d. in the dihedral angle between two l.s. planes) are estimated using the full covariance matrix. The cell e.s.d.'s are taken into account individually in the estimation of e.s.d.'s in distances, angles and torsion angles; correlations between e.s.d.'s in cell parameters are only used when they are defined by crystal symmetry. An approximate (isotropic) treatment of cell e.s.d.'s is used for estimating e.s.d.'s involving l.s. planes.
Refinement. Refinement of *F*^2^ against ALL reflections. The weighted *R*-factor *wR* and goodness of fit *S* are based on *F*^2^, conventional *R*-factors *R* are based on *F*, with *F* set to zero for negative *F*^2^. The threshold expression of *F*^2^ > σ(*F*^2^) is used only for calculating *R*-factors(gt) *etc*. and is not relevant to the choice of reflections for refinement. *R*-factors based on *F*^2^ are statistically about twice as large as those based on *F*, and *R*- factors based on ALL data will be even larger.

### Fractional atomic coordinates and isotropic or equivalent isotropic displacement parameters (Å^2^)

**Table:**

	*x*	*y*	*z*	*U*_iso_*/*U*_eq_	
O1	0.13353 (12)	−0.55544 (13)	0.41076 (5)	0.0504 (4)	
O2	0.01037 (12)	−0.45369 (13)	0.35657 (5)	0.0438 (3)	
O3	0.19132 (12)	0.35292 (11)	0.23292 (4)	0.0380 (3)	
O4	0.27173 (13)	0.23178 (11)	0.18499 (4)	0.0392 (3)	
O5	0.69961 (12)	0.11996 (11)	0.23969 (4)	0.0373 (3)	
O6	0.76216 (12)	0.23781 (11)	0.18596 (4)	0.0357 (3)	
O7	0.45898 (15)	1.00557 (18)	0.40087 (7)	0.0765 (6)	
O8	0.61390 (12)	0.93383 (14)	0.37529 (5)	0.0484 (4)	
N1	−0.04445 (12)	0.14840 (13)	0.40096 (5)	0.0265 (3)	
N2	0.13389 (12)	0.09436 (13)	0.45479 (5)	0.0254 (3)	
N3	0.62531 (13)	0.36677 (14)	0.45458 (5)	0.0310 (3)	
N4	0.44164 (12)	0.35297 (14)	0.39983 (5)	0.0302 (3)	
C1	0.10001 (14)	−0.03141 (15)	0.44837 (5)	0.0235 (3)	
C2	0.18112 (15)	−0.12287 (16)	0.46428 (5)	0.0273 (4)	
H2	0.2589	−0.1010	0.4782	0.033*	
C3	0.15049 (15)	−0.24462 (16)	0.46012 (6)	0.0295 (4)	
H3	0.2070	−0.3054	0.4715	0.035*	
C4	0.03710 (15)	−0.27907 (15)	0.43931 (5)	0.0275 (4)	
C5	0.00082 (16)	−0.41203 (16)	0.43529 (6)	0.0338 (4)	
H5A	0.0222	−0.4511	0.4664	0.041*	
H5B	−0.0857	−0.4167	0.4260	0.041*	
C6	0.05666 (15)	−0.48195 (15)	0.40050 (6)	0.0303 (4)	
C7	0.05890 (19)	−0.51676 (19)	0.32034 (7)	0.0463 (5)	
H7A	0.1425	−0.4968	0.3232	0.069*	
H7B	0.0172	−0.4911	0.2895	0.069*	
H7C	0.0498	−0.6050	0.3238	0.069*	
C8	−0.04269 (15)	−0.18824 (15)	0.42258 (6)	0.0269 (4)	
H8	−0.1198	−0.2108	0.4079	0.032*	
C9	−0.01339 (14)	−0.06501 (15)	0.42656 (5)	0.0244 (3)	
C10	−0.10157 (14)	0.03062 (16)	0.40602 (6)	0.0295 (4)	
H10A	−0.1428	0.0030	0.3749	0.035*	
H10B	−0.1608	0.0408	0.4266	0.035*	
C11	0.03016 (14)	0.17429 (15)	0.44624 (5)	0.0270 (4)	
H11A	−0.0161	0.1628	0.4716	0.032*	
H11B	0.0561	0.2605	0.4469	0.032*	
C12	0.21233 (14)	0.13466 (16)	0.42262 (5)	0.0266 (4)	
H12A	0.2771	0.0751	0.4234	0.032*	
H12B	0.2473	0.2146	0.4331	0.032*	
C13	0.14591 (14)	0.14595 (14)	0.37304 (5)	0.0238 (3)	
C14	0.20687 (15)	0.14763 (15)	0.33536 (6)	0.0271 (4)	
H14	0.2903	0.1503	0.3415	0.033*	
C15	0.14880 (16)	0.14550 (15)	0.28933 (6)	0.0291 (4)	
C16	0.21643 (19)	0.13771 (16)	0.24954 (6)	0.0378 (4)	
H16A	0.2977	0.1114	0.2623	0.045*	
H16B	0.1803	0.0737	0.2275	0.045*	
C17	0.22199 (15)	0.25389 (15)	0.22257 (6)	0.0276 (4)	
C18	0.2918 (2)	0.33655 (17)	0.15714 (6)	0.0427 (5)	
H18A	0.2164	0.3742	0.1439	0.064*	
H18B	0.3319	0.3106	0.1316	0.064*	
H18C	0.3409	0.3959	0.1770	0.064*	
C19	0.02681 (17)	0.14257 (15)	0.28116 (6)	0.0328 (4)	
H19	−0.0144	0.1401	0.2498	0.039*	
C20	−0.03572 (15)	0.14312 (15)	0.31745 (6)	0.0289 (4)	
H20	−0.1192	0.1415	0.3110	0.035*	
C21	0.02329 (14)	0.14598 (14)	0.36379 (5)	0.0242 (3)	
C22	0.50833 (15)	0.34330 (15)	0.36265 (6)	0.0270 (4)	
C23	0.44928 (17)	0.35394 (16)	0.31657 (6)	0.0333 (4)	
H23	0.3664	0.3645	0.3104	0.040*	
C24	0.51062 (19)	0.34919 (16)	0.27986 (6)	0.0387 (5)	
H24	0.4693	0.3571	0.2486	0.046*	
C25	0.63154 (19)	0.33299 (16)	0.28764 (6)	0.0363 (4)	
C26	0.6987 (2)	0.33876 (17)	0.24756 (7)	0.0483 (6)	
H26A	0.6562	0.3938	0.2233	0.058*	
H26B	0.7765	0.3758	0.2592	0.058*	
C27	0.71734 (15)	0.21890 (15)	0.22503 (6)	0.0284 (4)	
C28	0.79035 (17)	0.13024 (17)	0.16143 (6)	0.0359 (4)	
H28A	0.8519	0.0839	0.1816	0.054*	
H28B	0.8182	0.1545	0.1327	0.054*	
H28C	0.7200	0.0793	0.1533	0.054*	
C29	0.68939 (17)	0.31939 (16)	0.33344 (6)	0.0346 (4)	
H29	0.7719	0.3059	0.3392	0.042*	
C30	0.62957 (16)	0.32496 (15)	0.37123 (6)	0.0292 (4)	
C31	0.69570 (16)	0.31802 (18)	0.42110 (6)	0.0347 (4)	
H31A	0.7164	0.2320	0.4291	0.042*	
H31B	0.7697	0.3651	0.4237	0.042*	
C32	0.50945 (16)	0.30973 (17)	0.44408 (6)	0.0327 (4)	
H32A	0.5190	0.2201	0.4425	0.039*	
H32B	0.4657	0.3278	0.4698	0.039*	
C33	0.40789 (15)	0.47987 (17)	0.40761 (6)	0.0321 (4)	
H33A	0.3466	0.4803	0.4276	0.039*	
H33B	0.3741	0.5174	0.3771	0.039*	
C34	0.51138 (14)	0.55484 (16)	0.43082 (5)	0.0259 (3)	
C35	0.50775 (16)	0.68228 (16)	0.43139 (6)	0.0310 (4)	
H35	0.4371	0.7227	0.4180	0.037*	
C36	0.60485 (17)	0.75178 (16)	0.45100 (6)	0.0331 (4)	
C37	0.59910 (19)	0.88985 (18)	0.45326 (7)	0.0423 (5)	
H37A	0.5508	0.9130	0.4769	0.051*	
H37B	0.6794	0.9217	0.4640	0.051*	
C38	0.54884 (16)	0.95020 (16)	0.40758 (7)	0.0339 (4)	
C39	0.57179 (19)	0.9885 (2)	0.32980 (7)	0.0463 (5)	
H39A	0.4990	0.9480	0.3151	0.069*	
H39B	0.6314	0.9789	0.3098	0.069*	
H39C	0.5566	1.0755	0.3338	0.069*	
C40	0.70736 (16)	0.69204 (18)	0.47061 (6)	0.0347 (4)	
H40	0.7750	0.7381	0.4835	0.042*	
C41	0.71187 (15)	0.56696 (17)	0.47161 (6)	0.0317 (4)	
H41	0.7822	0.5274	0.4859	0.038*	
C42	0.61535 (14)	0.49713 (16)	0.45202 (5)	0.0265 (4)	

### Atomic displacement parameters (Å^2^)

**Table:**

	*U*^11^	*U*^22^	*U*^33^	*U*^12^	*U*^13^	*U*^23^
O1	0.0385 (8)	0.0433 (8)	0.0660 (10)	0.0107 (7)	0.0002 (7)	−0.0019 (7)
O2	0.0525 (8)	0.0448 (8)	0.0361 (7)	0.0137 (7)	0.0128 (6)	0.0003 (6)
O3	0.0568 (8)	0.0253 (7)	0.0353 (7)	0.0063 (6)	0.0175 (6)	0.0015 (5)
O4	0.0702 (9)	0.0274 (7)	0.0252 (6)	0.0035 (6)	0.0228 (6)	0.0034 (5)
O5	0.0562 (8)	0.0262 (7)	0.0315 (7)	−0.0006 (6)	0.0134 (6)	0.0014 (5)
O6	0.0555 (8)	0.0303 (7)	0.0248 (6)	0.0020 (6)	0.0164 (6)	−0.0003 (5)
O7	0.0632 (11)	0.0919 (14)	0.0805 (13)	0.0386 (10)	0.0294 (10)	0.0105 (10)
O8	0.0411 (8)	0.0682 (10)	0.0379 (8)	0.0125 (7)	0.0123 (6)	0.0044 (7)
N1	0.0246 (7)	0.0294 (8)	0.0263 (7)	0.0031 (6)	0.0070 (6)	−0.0007 (6)
N2	0.0263 (7)	0.0296 (7)	0.0208 (7)	−0.0020 (6)	0.0052 (5)	−0.0040 (6)
N3	0.0322 (8)	0.0376 (8)	0.0231 (7)	0.0030 (6)	0.0042 (6)	0.0037 (6)
N4	0.0303 (8)	0.0352 (8)	0.0256 (7)	−0.0058 (6)	0.0068 (6)	−0.0003 (6)
C1	0.0266 (8)	0.0306 (9)	0.0143 (7)	−0.0008 (7)	0.0064 (6)	−0.0020 (6)
C2	0.0259 (8)	0.0364 (10)	0.0196 (8)	0.0013 (7)	0.0038 (6)	−0.0010 (7)
C3	0.0338 (9)	0.0348 (10)	0.0208 (8)	0.0069 (7)	0.0077 (7)	0.0030 (7)
C4	0.0338 (9)	0.0309 (9)	0.0202 (8)	−0.0008 (7)	0.0106 (7)	0.0009 (7)
C5	0.0374 (10)	0.0328 (10)	0.0327 (10)	−0.0009 (8)	0.0103 (8)	0.0065 (8)
C6	0.0269 (9)	0.0229 (8)	0.0408 (10)	−0.0041 (7)	0.0052 (7)	0.0024 (7)
C7	0.0577 (13)	0.0399 (11)	0.0460 (12)	−0.0026 (10)	0.0218 (10)	−0.0110 (9)
C8	0.0253 (8)	0.0343 (9)	0.0222 (8)	−0.0036 (7)	0.0073 (6)	−0.0002 (7)
C9	0.0240 (8)	0.0315 (9)	0.0190 (8)	0.0003 (7)	0.0074 (6)	0.0007 (6)
C10	0.0236 (8)	0.0335 (9)	0.0322 (9)	0.0004 (7)	0.0071 (7)	0.0010 (7)
C11	0.0311 (9)	0.0292 (9)	0.0222 (8)	0.0024 (7)	0.0089 (7)	−0.0040 (7)
C12	0.0234 (8)	0.0325 (9)	0.0241 (8)	−0.0027 (7)	0.0053 (6)	−0.0036 (7)
C13	0.0290 (8)	0.0199 (8)	0.0230 (8)	−0.0013 (6)	0.0061 (6)	−0.0026 (6)
C14	0.0320 (9)	0.0228 (8)	0.0284 (9)	−0.0001 (7)	0.0103 (7)	−0.0014 (7)
C15	0.0468 (11)	0.0186 (8)	0.0246 (8)	0.0023 (7)	0.0143 (7)	0.0008 (6)
C16	0.0654 (13)	0.0253 (9)	0.0279 (9)	0.0061 (9)	0.0221 (9)	0.0025 (7)
C17	0.0374 (9)	0.0254 (9)	0.0201 (8)	0.0000 (7)	0.0048 (7)	−0.0002 (6)
C18	0.0682 (14)	0.0347 (10)	0.0287 (10)	−0.0001 (9)	0.0181 (9)	0.0093 (8)
C19	0.0506 (11)	0.0250 (9)	0.0210 (8)	0.0037 (8)	0.0014 (8)	−0.0005 (7)
C20	0.0314 (9)	0.0272 (9)	0.0266 (9)	0.0030 (7)	0.0008 (7)	−0.0001 (7)
C21	0.0281 (9)	0.0212 (8)	0.0238 (8)	0.0010 (6)	0.0055 (6)	−0.0008 (6)
C22	0.0358 (9)	0.0226 (8)	0.0230 (8)	−0.0041 (7)	0.0066 (7)	−0.0005 (6)
C23	0.0416 (10)	0.0288 (9)	0.0279 (9)	−0.0054 (8)	0.0016 (8)	−0.0009 (7)
C24	0.0649 (14)	0.0283 (9)	0.0218 (9)	−0.0065 (9)	0.0042 (9)	−0.0004 (7)
C25	0.0625 (13)	0.0212 (9)	0.0299 (9)	−0.0019 (8)	0.0210 (9)	−0.0011 (7)
C26	0.0887 (17)	0.0268 (10)	0.0377 (11)	−0.0040 (10)	0.0344 (11)	−0.0031 (8)
C27	0.0359 (9)	0.0290 (9)	0.0193 (8)	0.0002 (7)	0.0022 (7)	0.0003 (7)
C28	0.0433 (11)	0.0372 (10)	0.0274 (9)	0.0059 (8)	0.0071 (8)	−0.0058 (8)
C29	0.0457 (11)	0.0251 (9)	0.0363 (10)	0.0038 (8)	0.0160 (8)	−0.0013 (7)
C30	0.0373 (10)	0.0237 (8)	0.0276 (9)	0.0025 (7)	0.0085 (7)	0.0009 (7)
C31	0.0361 (10)	0.0388 (10)	0.0298 (9)	0.0101 (8)	0.0073 (8)	0.0021 (8)
C32	0.0405 (10)	0.0338 (10)	0.0253 (9)	−0.0031 (8)	0.0099 (7)	0.0047 (7)
C33	0.0231 (8)	0.0404 (10)	0.0330 (9)	−0.0012 (7)	0.0055 (7)	−0.0034 (8)
C34	0.0253 (8)	0.0346 (9)	0.0196 (8)	0.0003 (7)	0.0086 (6)	−0.0007 (7)
C35	0.0328 (9)	0.0374 (10)	0.0243 (9)	0.0045 (8)	0.0087 (7)	−0.0004 (7)
C36	0.0448 (11)	0.0359 (10)	0.0220 (8)	−0.0089 (8)	0.0150 (8)	−0.0067 (7)
C37	0.0552 (13)	0.0394 (11)	0.0344 (10)	−0.0064 (9)	0.0141 (9)	−0.0103 (8)
C38	0.0306 (9)	0.0269 (9)	0.0467 (11)	−0.0036 (7)	0.0136 (8)	−0.0095 (8)
C39	0.0546 (13)	0.0454 (12)	0.0392 (11)	0.0006 (10)	0.0091 (10)	0.0066 (9)
C40	0.0333 (10)	0.0501 (12)	0.0217 (8)	−0.0113 (8)	0.0075 (7)	−0.0067 (8)
C41	0.0269 (9)	0.0479 (11)	0.0206 (8)	−0.0020 (8)	0.0050 (7)	−0.0015 (7)
C42	0.0261 (8)	0.0372 (10)	0.0175 (8)	−0.0018 (7)	0.0078 (6)	0.0005 (7)

### Geometric parameters (Å, °)

**Table:**

O1—C6	1.197 (2)	C15—C19	1.389 (3)
O2—C6	1.328 (2)	C15—C16	1.504 (2)
O2—C7	1.449 (2)	C16—C17	1.501 (2)
O3—C17	1.196 (2)	C16—H16A	0.9900
O4—C17	1.340 (2)	C16—H16B	0.9900
O4—C18	1.445 (2)	C18—H18A	0.9800
O5—C27	1.195 (2)	C18—H18B	0.9800
O6—C27	1.341 (2)	C18—H18C	0.9800
O6—C28	1.443 (2)	C19—C20	1.377 (2)
O7—C38	1.189 (2)	C19—H19	0.9500
O8—C38	1.311 (2)	C20—C21	1.396 (2)
O8—C39	1.452 (2)	C20—H20	0.9500
N1—C21	1.438 (2)	C22—C23	1.394 (2)
N1—C11	1.466 (2)	C22—C30	1.394 (2)
N1—C10	1.469 (2)	C23—C24	1.379 (3)
N2—C1	1.436 (2)	C23—H23	0.9500
N2—C11	1.470 (2)	C24—C25	1.388 (3)
N2—C12	1.477 (2)	C24—H24	0.9500
N3—C42	1.434 (2)	C25—C29	1.385 (3)
N3—C32	1.461 (2)	C25—C26	1.507 (2)
N3—C31	1.471 (2)	C26—C27	1.499 (2)
N4—C22	1.434 (2)	C26—H26A	0.9900
N4—C32	1.460 (2)	C26—H26B	0.9900
N4—C33	1.472 (2)	C28—H28A	0.9800
C1—C2	1.394 (2)	C28—H28B	0.9800
C1—C9	1.401 (2)	C28—H28C	0.9800
C2—C3	1.380 (2)	C29—C30	1.395 (2)
C2—H2	0.9500	C29—H29	0.9500
C3—C4	1.395 (2)	C30—C31	1.514 (2)
C3—H3	0.9500	C31—H31A	0.9900
C4—C8	1.385 (2)	C31—H31B	0.9900
C4—C5	1.515 (2)	C32—H32A	0.9900
C5—C6	1.499 (2)	C32—H32B	0.9900
C5—H5A	0.9900	C33—C34	1.509 (2)
C5—H5B	0.9900	C33—H33A	0.9900
C7—H7A	0.9800	C33—H33B	0.9900
C7—H7B	0.9800	C34—C35	1.397 (2)
C7—H7C	0.9800	C34—C42	1.401 (2)
C8—C9	1.392 (2)	C35—C36	1.392 (2)
C8—H8	0.9500	C35—H35	0.9500
C9—C10	1.509 (2)	C36—C40	1.386 (3)
C10—H10A	0.9900	C36—C37	1.516 (3)
C10—H10B	0.9900	C37—C38	1.502 (3)
C11—H11A	0.9900	C37—H37A	0.9900
C11—H11B	0.9900	C37—H37B	0.9900
C12—C13	1.511 (2)	C39—H39A	0.9800
C12—H12A	0.9900	C39—H39B	0.9800
C12—H12B	0.9900	C39—H39C	0.9800
C13—C21	1.396 (2)	C40—C41	1.372 (3)
C13—C14	1.399 (2)	C40—H40	0.9500
C14—C15	1.384 (2)	C41—C42	1.389 (2)
C14—H14	0.9500	C41—H41	0.9500
			
C6—O2—C7	116.17 (15)	C20—C19—H19	119.2
C17—O4—C18	116.37 (14)	C15—C19—H19	119.2
C27—O6—C28	116.34 (14)	C19—C20—C21	120.10 (16)
C38—O8—C39	116.64 (16)	C19—C20—H20	120.0
C21—N1—C11	111.19 (13)	C21—C20—H20	120.0
C21—N1—C10	111.82 (13)	C13—C21—C20	119.59 (15)
C11—N1—C10	106.56 (13)	C13—C21—N1	121.58 (14)
C1—N2—C11	110.73 (13)	C20—C21—N1	118.83 (14)
C1—N2—C12	112.88 (12)	C23—C22—C30	119.43 (16)
C11—N2—C12	106.74 (13)	C23—C22—N4	118.41 (16)
C42—N3—C32	110.67 (14)	C30—C22—N4	122.15 (15)
C42—N3—C31	112.10 (14)	C24—C23—C22	120.27 (18)
C32—N3—C31	107.46 (14)	C24—C23—H23	119.9
C22—N4—C32	111.06 (14)	C22—C23—H23	119.9
C22—N4—C33	112.11 (13)	C23—C24—C25	121.25 (17)
C32—N4—C33	106.73 (14)	C23—C24—H24	119.4
C2—C1—C9	118.82 (15)	C25—C24—H24	119.4
C2—C1—N2	119.64 (14)	C29—C25—C24	118.23 (16)
C9—C1—N2	121.54 (14)	C29—C25—C26	121.02 (19)
C3—C2—C1	121.23 (16)	C24—C25—C26	120.63 (18)
C3—C2—H2	119.4	C27—C26—C25	115.57 (15)
C1—C2—H2	119.4	C27—C26—H26A	108.4
C2—C3—C4	120.42 (16)	C25—C26—H26A	108.4
C2—C3—H3	119.8	C27—C26—H26B	108.4
C4—C3—H3	119.8	C25—C26—H26B	108.4
C8—C4—C3	118.31 (16)	H26A—C26—H26B	107.4
C8—C4—C5	120.37 (15)	O5—C27—O6	123.78 (16)
C3—C4—C5	121.32 (16)	O5—C27—C26	126.39 (16)
C6—C5—C4	113.41 (14)	O6—C27—C26	109.79 (14)
C6—C5—H5A	108.9	O6—C28—H28A	109.5
C4—C5—H5A	108.9	O6—C28—H28B	109.5
C6—C5—H5B	108.9	H28A—C28—H28B	109.5
C4—C5—H5B	108.9	O6—C28—H28C	109.5
H5A—C5—H5B	107.7	H28A—C28—H28C	109.5
O1—C6—O2	123.48 (17)	H28B—C28—H28C	109.5
O1—C6—C5	124.39 (17)	C25—C29—C30	121.65 (18)
O2—C6—C5	112.12 (15)	C25—C29—H29	119.2
O2—C7—H7A	109.5	C30—C29—H29	119.2
O2—C7—H7B	109.5	C22—C30—C29	119.14 (16)
H7A—C7—H7B	109.5	C22—C30—C31	120.10 (15)
O2—C7—H7C	109.5	C29—C30—C31	120.69 (16)
H7A—C7—H7C	109.5	N3—C31—C30	111.47 (14)
H7B—C7—H7C	109.5	N3—C31—H31A	109.3
C4—C8—C9	122.04 (16)	C30—C31—H31A	109.3
C4—C8—H8	119.0	N3—C31—H31B	109.3
C9—C8—H8	119.0	C30—C31—H31B	109.3
C8—C9—C1	119.15 (15)	H31A—C31—H31B	108.0
C8—C9—C10	120.20 (15)	N4—C32—N3	112.08 (13)
C1—C9—C10	120.62 (15)	N4—C32—H32A	109.2
N1—C10—C9	111.41 (13)	N3—C32—H32A	109.2
N1—C10—H10A	109.3	N4—C32—H32B	109.2
C9—C10—H10A	109.3	N3—C32—H32B	109.2
N1—C10—H10B	109.3	H32A—C32—H32B	107.9
C9—C10—H10B	109.3	N4—C33—C34	111.82 (14)
H10A—C10—H10B	108.0	N4—C33—H33A	109.3
N1—C11—N2	111.73 (12)	C34—C33—H33A	109.3
N1—C11—H11A	109.3	N4—C33—H33B	109.3
N2—C11—H11A	109.3	C34—C33—H33B	109.3
N1—C11—H11B	109.3	H33A—C33—H33B	107.9
N2—C11—H11B	109.3	C35—C34—C42	118.10 (16)
H11A—C11—H11B	107.9	C35—C34—C33	121.76 (15)
N2—C12—C13	111.02 (13)	C42—C34—C33	120.14 (15)
N2—C12—H12A	109.4	C36—C35—C34	121.85 (17)
C13—C12—H12A	109.4	C36—C35—H35	119.1
N2—C12—H12B	109.4	C34—C35—H35	119.1
C13—C12—H12B	109.4	C40—C36—C35	118.64 (17)
H12A—C12—H12B	108.0	C40—C36—C37	119.64 (17)
C21—C13—C14	118.89 (15)	C35—C36—C37	121.66 (18)
C21—C13—C12	120.84 (14)	C38—C37—C36	114.47 (15)
C14—C13—C12	120.10 (14)	C38—C37—H37A	108.6
C15—C14—C13	121.73 (16)	C36—C37—H37A	108.6
C15—C14—H14	119.1	C38—C37—H37B	108.6
C13—C14—H14	119.1	C36—C37—H37B	108.6
C14—C15—C19	118.13 (15)	H37A—C37—H37B	107.6
C14—C15—C16	120.66 (17)	O7—C38—O8	123.1 (2)
C19—C15—C16	121.08 (16)	O7—C38—C37	124.11 (18)
C17—C16—C15	115.23 (14)	O8—C38—C37	112.75 (16)
C17—C16—H16A	108.5	O8—C39—H39A	109.5
C15—C16—H16A	108.5	O8—C39—H39B	109.5
C17—C16—H16B	108.5	H39A—C39—H39B	109.5
C15—C16—H16B	108.5	O8—C39—H39C	109.5
H16A—C16—H16B	107.5	H39A—C39—H39C	109.5
O3—C17—O4	123.79 (15)	H39B—C39—H39C	109.5
O3—C17—C16	126.69 (16)	C41—C40—C36	120.49 (17)
O4—C17—C16	109.48 (14)	C41—C40—H40	119.8
O4—C18—H18A	109.5	C36—C40—H40	119.8
O4—C18—H18B	109.5	C40—C41—C42	121.11 (17)
H18A—C18—H18B	109.5	C40—C41—H41	119.4
O4—C18—H18C	109.5	C42—C41—H41	119.4
H18A—C18—H18C	109.5	C41—C42—C34	119.77 (16)
H18B—C18—H18C	109.5	C41—C42—N3	118.42 (15)
C20—C19—C15	121.52 (16)	C34—C42—N3	121.82 (15)
			
C11—N2—C1—C2	−166.37 (14)	C32—N4—C22—C23	165.62 (15)
C12—N2—C1—C2	74.03 (18)	C33—N4—C22—C23	−75.07 (19)
C11—N2—C1—C9	13.35 (19)	C32—N4—C22—C30	−15.0 (2)
C12—N2—C1—C9	−106.24 (16)	C33—N4—C22—C30	104.28 (18)
C9—C1—C2—C3	−1.9 (2)	C30—C22—C23—C24	−1.5 (3)
N2—C1—C2—C3	177.80 (14)	N4—C22—C23—C24	177.88 (15)
C1—C2—C3—C4	0.7 (2)	C22—C23—C24—C25	0.4 (3)
C2—C3—C4—C8	0.8 (2)	C23—C24—C25—C29	1.2 (3)
C2—C3—C4—C5	−178.71 (15)	C23—C24—C25—C26	−174.72 (16)
C8—C4—C5—C6	109.90 (18)	C29—C25—C26—C27	91.4 (2)
C3—C4—C5—C6	−70.6 (2)	C24—C25—C26—C27	−92.9 (2)
C7—O2—C6—O1	−0.5 (3)	C28—O6—C27—O5	−0.5 (3)
C7—O2—C6—C5	179.95 (15)	C28—O6—C27—C26	177.14 (16)
C4—C5—C6—O1	106.9 (2)	C25—C26—C27—O5	−11.5 (3)
C4—C5—C6—O2	−73.57 (19)	C25—C26—C27—O6	170.94 (17)
C3—C4—C8—C9	−0.9 (2)	C24—C25—C29—C30	−1.8 (3)
C5—C4—C8—C9	178.58 (15)	C26—C25—C29—C30	174.11 (16)
C4—C8—C9—C1	−0.4 (2)	C23—C22—C30—C29	0.9 (2)
C4—C8—C9—C10	177.43 (15)	N4—C22—C30—C29	−178.45 (15)
C2—C1—C9—C8	1.8 (2)	C23—C22—C30—C31	177.67 (16)
N2—C1—C9—C8	−177.96 (14)	N4—C22—C30—C31	−1.7 (2)
C2—C1—C9—C10	−176.02 (14)	C25—C29—C30—C22	0.7 (3)
N2—C1—C9—C10	4.2 (2)	C25—C29—C30—C31	−176.02 (16)
C21—N1—C10—C9	71.68 (17)	C42—N3—C31—C30	−72.47 (19)
C11—N1—C10—C9	−50.01 (17)	C32—N3—C31—C30	49.33 (19)
C8—C9—C10—N1	−162.57 (14)	C22—C30—C31—N3	−16.3 (2)
C1—C9—C10—N1	15.2 (2)	C29—C30—C31—N3	160.41 (16)
C21—N1—C11—N2	−50.30 (18)	C22—N4—C32—N3	51.30 (19)
C10—N1—C11—N2	71.78 (16)	C33—N4—C32—N3	−71.18 (17)
C1—N2—C11—N1	−51.81 (17)	C42—N3—C32—N4	52.55 (18)
C12—N2—C11—N1	71.41 (16)	C31—N3—C32—N4	−70.14 (18)
C1—N2—C12—C13	72.00 (17)	C22—N4—C33—C34	−72.87 (18)
C11—N2—C12—C13	−49.88 (16)	C32—N4—C33—C34	48.95 (17)
N2—C12—C13—C21	14.0 (2)	N4—C33—C34—C35	164.18 (15)
N2—C12—C13—C14	−161.30 (14)	N4—C33—C34—C42	−14.9 (2)
C21—C13—C14—C15	−2.0 (2)	C42—C34—C35—C36	2.0 (2)
C12—C13—C14—C15	173.41 (15)	C33—C34—C35—C36	−177.16 (15)
C13—C14—C15—C19	0.5 (2)	C34—C35—C36—C40	−0.3 (2)
C13—C14—C15—C16	−175.30 (15)	C34—C35—C36—C37	−177.57 (16)
C14—C15—C16—C17	−105.8 (2)	C40—C36—C37—C38	132.60 (18)
C19—C15—C16—C17	78.5 (2)	C35—C36—C37—C38	−50.2 (2)
C18—O4—C17—O3	2.3 (3)	C39—O8—C38—O7	0.7 (3)
C18—O4—C17—C16	−175.43 (16)	C39—O8—C38—C37	179.70 (16)
C15—C16—C17—O3	9.8 (3)	C36—C37—C38—O7	114.6 (2)
C15—C16—C17—O4	−172.60 (16)	C36—C37—C38—O8	−64.4 (2)
C14—C15—C19—C20	0.7 (2)	C35—C36—C40—C41	−1.5 (2)
C16—C15—C19—C20	176.50 (15)	C37—C36—C40—C41	175.78 (16)
C15—C19—C20—C21	−0.4 (3)	C36—C40—C41—C42	1.7 (3)
C14—C13—C21—C20	2.4 (2)	C40—C41—C42—C34	0.0 (2)
C12—C13—C21—C20	−173.05 (15)	C40—C41—C42—N3	−179.44 (15)
C14—C13—C21—N1	−177.90 (14)	C35—C34—C42—C41	−1.8 (2)
C12—C13—C21—N1	6.7 (2)	C33—C34—C42—C41	177.34 (15)
C19—C20—C21—C13	−1.2 (2)	C35—C34—C42—N3	177.64 (14)
C19—C20—C21—N1	179.04 (15)	C33—C34—C42—N3	−3.2 (2)
C11—N1—C21—C13	10.9 (2)	C32—N3—C42—C41	164.77 (14)
C10—N1—C21—C13	−108.06 (17)	C31—N3—C42—C41	−75.28 (18)
C11—N1—C21—C20	−169.33 (14)	C32—N3—C42—C34	−14.7 (2)
C10—N1—C21—C20	71.69 (18)	C31—N3—C42—C34	105.26 (17)
